# The response of Arctic vegetation and soils following an unusually severe tundra fire

**DOI:** 10.1098/rstb.2012.0490

**Published:** 2013-08-19

**Authors:** M. Syndonia Bret-Harte, Michelle C. Mack, Gaius R. Shaver, Diane C. Huebner, Miriam Johnston, Camilo A. Mojica, Camila Pizano, Julia A. Reiskind

**Affiliations:** 1 Institute of Arctic Biology, University of Alaska, Fairbanks, AK 99775, USA; 2 Department of Botany, University of Florida, Gainesville, FL 32611, USA; 3 The Ecosystems Center, Marine Biological Laboratory, Woods Hole, MA 02543, USA

**Keywords:** Alaskan tussock tundra, fire, vegetation recovery, permafrost, climate change, soil N availability

## Abstract

Fire causes dramatic short-term changes in vegetation and ecosystem function, and may promote rapid vegetation change by creating recruitment opportunities. Climate warming likely will increase the frequency of wildfire in the Arctic, where it is not common now. In 2007, the unusually severe Anaktuvuk River fire burned 1039 km^2^ of tundra on Alaska's North Slope. Four years later, we harvested plant biomass and soils across a gradient of burn severity, to assess recovery. In burned areas, above-ground net primary productivity of vascular plants equalled that in unburned areas, though total live biomass was less. Graminoid biomass had recovered to unburned levels, but shrubs had not. Virtually all vascular plant biomass had resprouted from surviving underground parts; no non-native species were seen. However, bryophytes were mostly disturbance-adapted species, and non-vascular biomass had recovered less than vascular plant biomass. Soil nitrogen availability did not differ between burned and unburned sites. Graminoids showed allocation changes consistent with nitrogen stress. These patterns are similar to those seen following other, smaller tundra fires. Soil nitrogen limitation and the persistence of resprouters will likely lead to recovery of mixed shrub–sedge tussock tundra, unless permafrost thaws, as climate warms, more extensively than has yet occurred.

## 1. Introduction

Climate change is occurring rapidly at high latitudes, where surface air temperature has increased at twice the rate of the rest of the globe in the past decades [1,2]. During this period, the frequency of large fires in the boreal forest has also increased dramatically [3]. Wildfires are presently uncommon in Arctic tundra ecosystems in northern Alaska, except for limited areas in the Noatak watershed and the Seward Peninsula [4], but are expected to become more common as climate warms [1,2,5]. Wildfires can have strong ecosystem consequences for both the radiation and carbon balance of tundra. By combusting soil organic matter and vegetation, fire can rapidly transfer large stocks of soil carbon to the atmosphere, reducing C storage and changing the C balance of ecosystems in the short-term [6,7]. Fire in tundra ecosystems also darkens the surface and removes some, or all, of the insulating layer of moss and soil organic matter that shields the underlying permafrost from warm summer temperatures. Thus, fire changes the surface radiation balance and may lead to destabilization of deeper stores of soil C, resulting in additional C transfer to the atmosphere long after the initial combustion. Soils underlain by permafrost in Arctic and boreal regions are estimated to hold nearly twice the total amount of C as is in the atmosphere [8], so increased fire frequency in tundra may alter the magnitude of Arctic feedbacks to global climate that are already occurring in response to climate warming.

In addition to these direct effects, wildfire changes the composition and structure of vegetation in the short-term, and in the long-term if wildfire alters the successional trajectory. Vegetation composition affects both ecosystem C balance and fire frequency, because some types of vegetation are more flammable than others [7]. In addition, a change in successional trajectory may result in the ecosystem undergoing a regime shift into an alternate state, in which the structure of the vegetation and ecosystem function is altered, and ecosystem services may be degraded [9]. In most small tundra fires that have been studied so far, vegetation cover has recovered within 5–10 years, though there may be transient changes in relative abundance of different functional groups of plants for the first decade, and some subtle changes in composition may persist for several decades [10–15]. However, in at least three high-severity tundra fires, vegetation has shifted towards dominance by deciduous shrubs [11,16] or grasses [17], which have persisted for more than 30 years and may represent regime shifts. A widespread increase in the cover of large deciduous shrubs appears to be underway in the Arctic [18,19], coincident with recent warming. If fires promote development of shrub-dominated vegetation under a warming climate, then this might positively feed back to more frequent fires: palaeorecords indicate that ancient shrub-dominated tundra burned as often as modern boreal forest, in part by creating an abundance of fine, flammable fuels [20]. However, the factors that control successional trajectories following fire in tundra vegetation are not well understood.

Soil nitrogen availability may be one important factor controlling the successional trajectory of vegetation following fire. Above-ground net primary production (ANPP) in tussock tundra is strongly nutrient-limited, and deciduous shrubs respond positively to increased soil nutrient availability in long-term warming and fertilization experiments in tussock tundra [21–24]. If fire promotes increased soil N availability, then it may hasten the conversion to shrub-dominated vegetation. Conversely, if fire depletes soil N stocks through combustion and post-fire soil N availability is reduced, then this may limit productivity and constrain the successional trajectory back towards tussock tundra. In northern coniferous forests, severe fires are variable across space and time, and ecologists have observed differing patterns of post-fire soil N availability [25]. At present, it is not clear how severe fires in tundra ecosystems may alter soil N availability.

The Anaktuvuk River fire of 2007 was unprecedented in size (1039 km^2^) and severity for a fire on the 188 448 km^2^ Alaskan North Slope within the historical record [6,26,27]. This fire doubled the cumulative area of tundra in this region that had burned in the past 50 years [6]. Remotely sensed indices of burn severity were substantially higher than for any other tundra fire [26], and it burned in an area with no evidence of fires for at least 5000 years, based on charcoal deposition in lake-sediment cores [27]. A combination of unusually warm and dry conditions associated with a late-season high-pressure system located over the Beaufort Sea, and possibly linked to sea ice retreat [27], are thought to be responsible for its size and severity. Combustion released an estimated 2.1 Tg of carbon to the atmosphere, an amount similar to the annual ANPP by the entire tundra biome worldwide [6]. An estimated 37 years of C accumulation, but nearly 400 years of N accumulation, was combusted in the fire [6].

Our goal in this study was to assess vegetation recovery and N dynamics in vegetation and soils 4 years after the Anaktuvuk River fire, across a gradient of burn severity, using sites in nearby unburned tundra as a proxy for pre-fire vegetation and soils. We also wished to assess whether post-fire succession may result in mixed shrub–sedge tussock tundra vegetation similar to what was present before the fire, or whether this area could be on a new successional trajectory to a different vegetative state. Possible alternative vegetative states could include persistent dominance by deciduous shrubs, persistent dominance by grasses or introduction of novel species resulting in a vegetation composition that does not currently exist on Alaska's North Slope. Although our measurements were made at only one point in time, our data and comparisons with the results of prior studies on other tundra fires allow inferences about successional trajectories to be made.

## 2. Material and methods

### (a) Site description

To measure recovery of vegetation and soils 4 years after the fire, in July 2011, we harvested biomass and soils along six 50-m-long transects located either within or near the southeastern portion of the Anaktuvuk River fire scar (table 1), roughly 37 km NW of the Toolik Field Station (68.583° N, 149.717° W), which is the site of the Arctic Long-Term Ecological Research (LTER) programme. The number and location of transects that could be harvested was restricted by the need to access sites by helicopter, and the time required to collect samples in the field. Harvest transects were in unburned areas observed, or burned areas inferred (before the fire), to consist of acidic mixed shrub–sedge tussock tundra vegetation [28,29], and were located immediately adjacent to a subset of transects established in 2008 for repeated non-destructive monitoring of vegetation cover along several toposequences [30]. The harvested transects had similar vegetation composition to that of other transects established in moist acidic tundra in the foothills region of the burn scar [30], and thus were felt to be representative. Prior to the fire, 54 per cent of the entire area burned in the Anaktuvuk River fire was classified as upland moist acidic tundra (soil pH < 5.5), 15 per cent as moist non-acidic tundra (pH > 5.5) and 30 per cent as shrubland [30]. In an unburned state, moist acidic tussock tundra contains approximately equal biomass of graminoids (primarily *Eriophorum vaginatum* and *Carex bigelowii*), deciduous shrubs (*Betula nana*, *Salix pulchra*, *Salix glauca* and *Vaccinium uliginosum*), evergreen shrubs (mainly *Ledum palustre* ssp. *decumbens* and *Vaccinium vitis-idaea*) and mosses (*Hylocomium splendens*, *Aulacomnium turgidum, Dicranum* spp*., Sphagnum* spp., etc.) [31]. The mean pH of organic soils from our harvest transects ranged from 4.4 to 4.9 (table 1).

**Table 1.** Locations and burn severity for transects sampled for this study. Coordinates (in decimal degrees) were obtained in the North American Datum of 1983. Burn severity scores are for adjacent permanent transects that were scored in 2008, the first summer after the fire.

permanent transect ID	latitude of origin (° N)	longitude of origin (° W)	elevation (m)	burn severity class	burn severity score	soil pH (± s.e.)
101	69.995	150.283	359	severe	1.8	4.6 (0.18)
114	69.997	150.307	325	severe	1.5	4.5 (0.08)
103	68.954	150.207	403	moderate	3.1	4.7 (0.05)
104	68.951	150.210	412	moderate	2.5	4.9 (0.12)
108	68.952	150.208	414	unburned	4.8	4.4 (0.15)
109	68.933	150.273	435	unburned	5	4.5 (0.10)

In the first summer after the fire (July 2008), burn severity was assessed for vegetation and soils in 10 quadrats (1×1 m) located at 5-m intervals along each non-destructive transect, using a scale of 1 (heavily burned) to 5 (unburned), based on damage to vegetation and surface soils, according to the Alaska Interagency Fire Effects Task Group protocol [32]. Of the six non-destructive transects adjacent to transects harvested in 2011, two were classified as unburned (average burn severity 4.9), two as moderately burned (average burn severity 2.8) and two as severely burned (average burn severity 1.6; table 1). From satellite imagery, 39–43% of the entire fire scar was classified as moderate-to-low severity, 47–59% was classified as high severity and 2–11% was classified as unburned [26,33]; this was unusually severe for a tundra fire. Although classification of the entire fire scar was at a large-scale, the plot-level classification is reasonably consistent with classes used for satellite imagery [30].

### (b) Biomass and soil harvest

Biomass and soil were harvested from 23 to 25 July 2011 in a stratified random manner. For each harvest transect, one random number was selected within each of the 10 contiguous 5-m intervals, and biomass was harvested from a 10 × 40 cm quadrat located at each random point. This ensured that the 10 locations harvested from each transect were distributed along its entire length. Biomass was harvested using previously described methods [31,34]. Briefly, the rhizome-containing soil layer was harvested by cutting around each quadrat boundary with a serrated bread knife. All above-ground live vascular plant biomass as well as all live rhizomes and below-ground stems within the quadrat boundaries were separated by species. Current year's growth from meristems located within the quadrat was included in the sample even if that growth extended outside the quadrat. New growth from meristems located outside, but that extended into the quadrat, was not included in the sample. For older stems that crossed the boundary, only the portion within the quadrat boundaries was included.

The current year's growth from each vascular plant was separated into leaves, new above-ground stems, and inflorescences with their peduncles, except that new growth from rhizomes was included with previous years' growth. Older biomass was separated into (i) below-ground stems and rhizomes, (ii) above-ground stems, and (iii) old leaves (for evergreens only). All graminoid, forb and deciduous shrub leaves were considered to be new biomass. Below-ground stems were separated from above-ground old stems at the position of the first adventitious roots. Vascular plant litter and attached dead biomass were saved, but not separated by species. These biomass harvest methods are consistent with our previous and ongoing LTER studies and thus allow direct comparisons [22,31,34].

Lichens, mosses (green portions only) and liverworts (*Marchantia polymorpha* from burned sites) were not separated into species or new and old growth. Green moss biomass has been estimated to consist of approximately 20 per cent new growth in those species where old and new growth can be distinguished, but they are not the majority of moss species present in tussock tundra [21]. All plant samples were dried at 60°C for 72 h and weighed.

We calculated ANPP for vascular plants as the sum of the current year's primary growth (new growth samples mentioned above), plus stem secondary growth for the three largest shrub species (*S. pulchra, B. nana* and *L. palustre*). Secondary growth was calculated from old stem biomass, and relative secondary growth rates determined in a previous study at Toolik Lake [35]. As we did not have reliable measures of their growth, ANPP was not calculated for non-vascular plants.

To determine soil bulk density, element concentration and root biomass, organic soil horizons were sampled volumetrically with a serrated knife. From every other point on the transect where a quadrat was harvested for biomass determinations (five locations per transect), two contiguous 10 × 20 cm soil monoliths were excised from the side of the pit, extending from the surface of the green moss to the surface of the mineral soil (roughly 5–30 cm depth depending on location). In addition, two cores (2.42 cm in diameter) were taken through the mineral soil from the bottom of these monoliths to the ice surface at the bottom of the thawed layer. Monoliths and cores were wrapped in tinfoil to preserve structure, returned to the field station and refrigerated prior to analyses. Within 24 h of collection, one monolith and one core from each location were processed to determine microbial biomass, bulk density, moisture and C and N concentrations in both soluble (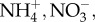 dissolved organic N (DON) and dissolved organic C (DOC)) pools and solid fractions. Coarse (more than 2 mm in diameter) and fine (less than 2 mm in diameter) roots were separated by hand from the other monolith and core. Fine roots were separated into *E. vaginatum* roots (which can be distinguished owing to their vertical growth pattern, consistent diameter and lack of branching) and other roots.

### (c) Carbon and nitrogen content analyses

We pooled material of each plant tissue type (excluding roots) and species from all quadrats from a given transect. Pooled samples were ground in a Wiley mill with a 40 mm sieve and analysed for C and N content by a ThermoScientific Flash 2000 NC soil analyser (ThermoFisher Scientific, Cambridge, UK). C and N pools in biomass and ANPP were calculated by multiplying the biomass of each plant species and tissue component by the appropriate concentration of C or N (from the pooled sample), and then summing over all species and tissues for each growth form in each quadrat. Roots were processed separately for each sampled quadrat. Root samples were analysed for C and N content on a Costech elemental analyser (Costech Analytical, Los Angeles, CA) calibrated with the NIST peach leaves standard (SRM 1547, National Institute of Standards and Technology, Gaithersburg, MD).

Soil samples from monoliths and cores were homogenized by hand, and coarse organic materials (twigs and roots more than 2.5 cm in diameter) and rocks were removed. A subsample of 10 g (wet weight) of homogenized soil was extracted with 2 M KCl overnight, for determination of soluble DOC, DON, 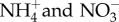 concentrations. A second 10 g subsample was fumigated with chloroform for 24 h prior to extraction with 2 M KCl as above, for determination of microbial biomass N [36]. Extracts were frozen prior to analysis for 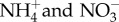 using an Astoria Pacific (Astoria, OR) colorimetric autoanalyser, and a Shimadzu TOC-L autoanalyser with a TN unit (Columbia, MO) for DOC and DON. Coarse and fine soil fractions were weighed wet, dried at 70°C for 48 h and reweighed for dry matter content, then ground on a Wiley mill with a 40 mm sieve. Dry matter content of fine mineral soil was determined on subsamples dried at 105°C for 48 h. For all samples dried at 70°C, C and N content was measured on the same Costech elemental analyser as the roots. The volume of each organic monolith layer or mineral core was calculated as depth times area minus the volume of rocks. Bulk density, C and N pools for soil fractions, and C and N pools for extracted fractions were calculated for both mineral and organic horizons.

### (d) Statistical analyses

All data are available from the website of the Arctic LTER at http://dryas.mbl.edu/ARC/datacatalog.html. All plant and soil variables were analysed by nested ANOVA (generalized linear model with burn severity as the main factor and transect nested within burn severity; JMP Statistical Software). The nested term pools the variance associated with transect and with transect by burn severity interaction, and is appropriate because each individual transect occurred within only one burn severity level [37]. For most variables, the transect term was not significant, but partitioned away variance associated with transect, enabling the effect of burn severity to become more apparent. All data were tested for homogeneity of variance prior to analysis using Levene, Bartlett, O'Brien and Brown–Forsythe tests [37]. If two or more tests did not indicate homogeneity of variance, then data were either transformed or ranked (using the average of tied ranks) prior to ANOVA (same model as above). ANOVA was conducted on ranks only if transformation by the algorithms *y* = ln(*x* + 1) or 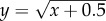 did not result in homogeneity of variance [38]. In two cases, no transformation was successful in achieving homogeneity of variance; here, ANOVA was performed on untransformed data, and noted in the footnote to the table.

ANOVAs for plant variables excluding roots used the full dataset (10 quadrats per transect). Analyses of soil variables, roots and variables that combined roots with above-ground biomass used only values from locations where soils and roots were sampled with above-ground plant biomass (five quadrats per transect). Many species occurred so rarely that their biomass data could not be analysed separately, particularly in burned transects. Biomass was variable even for common species because of the heterogeneity of vegetation at the scale of a 10 × 40 cm quadrat. Accordingly, we present statistical tests mainly for growth forms. All species present were included within the summed data for their growth form. If a tissue type, species or growth form was absent from a particular quadrat, its biomass or N pool value was zero; because C : N was undefined in this case, these quadrats were not included in these ANOVAs, and there were fewer degrees of freedom than for ANOVAs of biomass or N pools.

Although the number of transects within each burn severity class was small, many differences among burn severity classes were statistically significant. Thus, our sampling design had sufficient power to detect differences in these variables. Post-experiment power analyses are not useful for interpreting non-significant results [39]. For most variables that were not significantly different, there was high variability and no consistent trend between burned and unburned sites. Thus, these data do not suggest that a significant difference would have been detected with a larger sample size, although the possibility of type II error cannot be excluded. Differences that could not be detected with the sampling design would be small compared with those that were detected, and thus are unlikely to explain the patterns that we saw.

## 3. Results

### (a) Live plant biomass and ANPP

**Figure 1.**
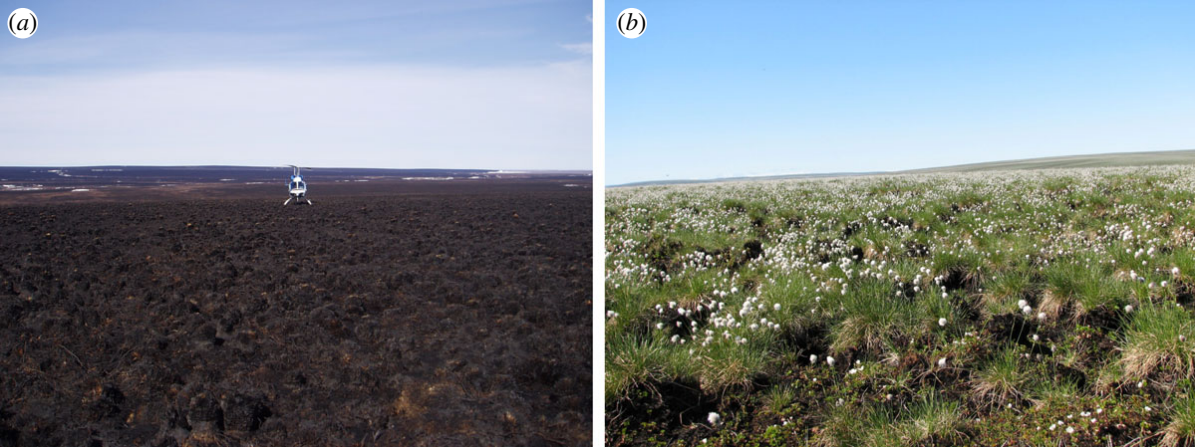
Representative tussock tundra within the area burned by the Anaktuvuk River fire: (*a*) in June 2008, the first summer after the fire, and (*b*) in July 2010. Note the resprouting tussocks of *Eriophorum vaginatum* that were present before the fire. (Online version in colour.)

**Figure 2.**
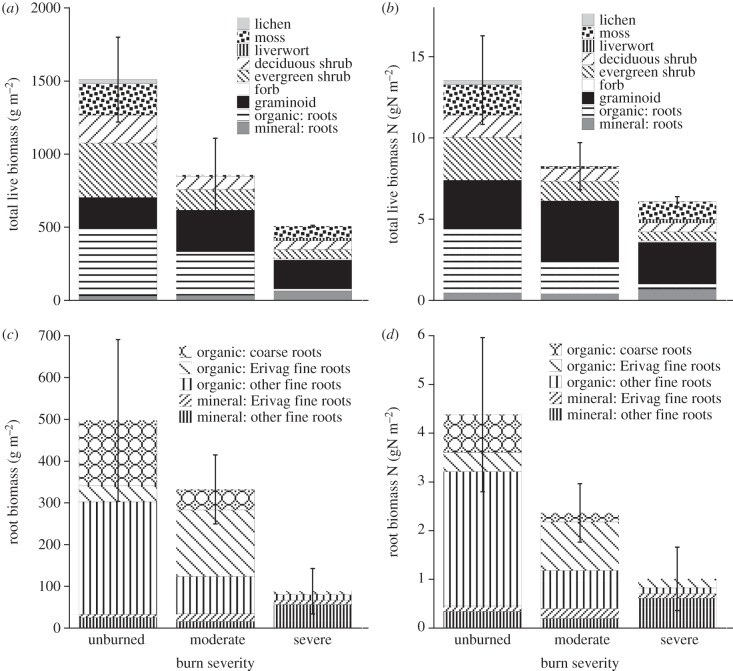
Biomass and N content in plants by growth form (lichen, moss, liverworts, deciduous shrubs, evergreen shrubs and graminoids) from unburned, moderately burned and severely burned transects in July 2011. (*a*) Total biomass including roots. Roots from the mineral and organic soil layers are indicated separately, and are not separated by growth form. (*b*) Mass of N in living biomass, with the same separation as in (*a*). (*c*) Living root biomass showing the separation into coarse and fine roots, which are further divided into fine roots of *E. vaginatum* (Erivag) and other fine roots. (*d*) Mass of N in living roots, divided as in (*c*). Error bars = 1 s.e. for the total community biomass, N mass, root biomass, and root biomass N, between transects (*n* = 2).

Four years after the Anaktuvuk River fire, vascular tundra vegetation had begun to recover, but had not reached pre-fire levels (figures 1 and 2). Although total plant biomass was still significantly greater in unburned transects than in burned transects, graminoid biomass was not significantly different (figure 2*a* and table 2). This was largely due to resprouting by tillers of the tussock-forming sedge *E. vaginatum* that had survived the fire, because their living rhizomes and meristems were protected by being enclosed in a dense, moist column of dead leaf bases, rhizomes and roots (the tussock core), even though all their leaves were killed in the fire (figure 1). By contrast, although deciduous and evergreen shrubs had resprouted from surviving subterranean stems, their total biomass remained significantly below that of unburned sites (figure 2*a* and table 2), because all of their above-ground living biomass had been killed or consumed by the fire. The biomass of evergreen shrubs was significantly lower in severely burned transects than in moderately burned transects, which was not true of any other plant growth form. Forb biomass did not differ significantly between burned and unburned sites, but forb biomass was not captured well at the scale of our harvest, being low (means were less than 2% of live biomass) and variable (figure 2*a* and table 2). All vascular plant species found in burned transects were also found in unburned transects, and virtually all the biomass in burned transects was made up of resprouting individuals that had survived the fire. Although numerous seedlings occurred in the burn, their biomass was negligible.

**Table 2.** Results of analysis of variance on live plant biomass by growth form, and on live roots. Treatments that share the same lower case letter were not significantly different in post-hoc tests (Tukey's HSD) performed when burn severity was significant at *p* < 0.05 (bold). Ndf, numerator degrees of freedom; Ddf, denominator degrees of freedom; U, unburned; M, moderately burned; S, severely burned.

growth form/category	factor								
	burn severity					transect [burn severity]			
	Ndf	Ddf	*F*	*p*	post-hoc	Ndf	Ddf	*F*	*p*
plant biomass excluding roots^a^	2	54	23.6959	**<0.0001**	U*a*; > M*b*; S*b*	3	54	1.0499	0.3781
graminoids	2	54	0.7115	0.4955		3	54	0.5852	0.6273
deciduous shrubs	2	54	7.5593	**0.0013**	U*a*; > M*b*; S*b*	3	54	0.4007	0.7530
evergreen shrubs^c^	2	54	28.7409	**<0.0001**	U*a*; > M*b*; > S*c*	3	54	1.6184	0.1958
forbs	2	54	1.0977	0.3354		3	54	0.8577	0.4638
all roots^b^*^,^*^c^	2	24	12.1941	**0.0002**	U*a*; M*a*; > S*b*	3	24	2.2776	0.1053
all roots from organic soil^b,c^	2	24	26.6855	**<0.0001**	U*a*; M*a*; > S*b*	3	24	2.4605	0.0871
fine roots from organic soil^c^	2	24	23.6155	**<0.0001**	U*a*; M*a*; > S*b*	3	24	1.9694	0.1455
*Eriophorum* roots^c^	2	24	5.4670	**0.0111**	U*ab*; M*a*; > S*b*	3	24	0.9743	0.4212
other roots^c^	2	24	29.4634	**<0.0001**	U*a*; > M*b*; > S*c*	3	24	0.3440	0.7938
fine roots from mineral soil	2	24	0.8360	0.4457		3	24	1.5948	0.2167
*Eriophorum* roots	2	24	1.1126	0.3451		3	24	0.6389	0.5974
other roots^c^	2	24	1.0578	0.3628		3	24	1.1856	0.3362
all plant biomass including roots	2	24	19.7349	**<0.0001**	U*a*; > M*b*; > S*c*	3	24	1.8475	0.1655

^a^Includes data on moss and lichen biomass, which could not be analysed separately because they did not meet the assumptions of analysis of variance.

^b^Includes coarse roots from organic soil, which could not be analysed separately because there were no coarse roots in the severely burned transects.

^c^Data were rank-transformed to meet the assumptions of homogeneity of variance.

Total live root biomass did not differ significantly between unburned and moderately burned transects, but was significantly lower in severely burned transects (figure 2*a,c* and table 2). In the soil organic layer, coarse roots (more than 2 mm) of shrubs were not found at all in severely burned transects, and were lower in moderately burned transects than in unburned transects (figure 2*c*), although their occurrence was so patchy that it could not be analysed by ANOVA. No coarse roots occurred in mineral soil. It was not feasible to separate fine roots by species, other than those of *E. vaginatum*. Most root biomass in the moderately burned sites was composed of fine *E. vaginatum* roots in the organic soil layer, which were higher (though not significantly) in moderately burned than in unburned transects. Biomass of other fine roots in organic soil decreased significantly from unburned to moderately burned to severely burned transects, and there were no significant differences in fine root biomass in the mineral soil horizon (figure 2*c* and table 2).

The difference in both biomass and plant composition between burned and unburned sites was much greater for non-vascular than for vascular plants (figure 2*a*). No live lichens were found in any burned transect, and many burned quadrats had no live moss biomass. Some severely burned transects on mineral soil supported a lush carpet of the liverwort *M. polymorpha* (commonly seen in boreal forests after fire) and ‘weedy’ mosses, including *Polytrichum* spp., *Ceratodon purpureus* and *Pohlia nutans*. The moss community in unburned sites was dominated by *Sphagnum* spp., with abundant *Aulacomnium turgidum* and *H. splendens*, but no *Sphagnum* was seen in burned sites. *Aulacomnium turgidum* was found in both burned and unburned sites, but was much less common in burned sites. Because of the patchy occurrence of non-vascular plants in the burned transects, their biomass data could not be analysed by ANOVA.

**Figure 3.**
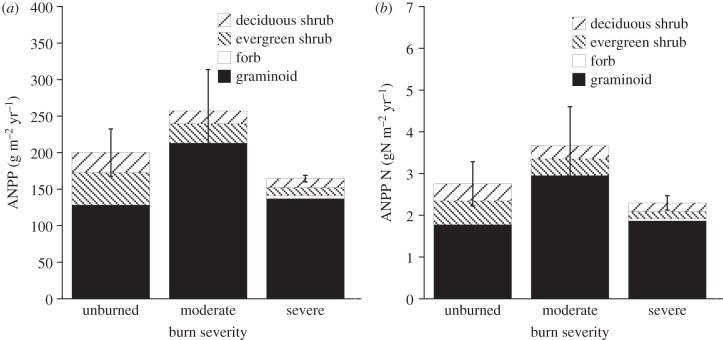
(*a*) Above-ground net primary productivity (ANPP) for vascular plants, by growth form. (*b*) Mass of N in ANPP, by growth form. Error bars = 1 s.e. for total community ANPP or mass of N in ANPP, between transects (*n* = 2).

In contrast to biomass, ANPP of vascular plants did not differ significantly between burned and unburned transects (figure 3 and table 3). Burn severity did not affect ANPP of either graminoids or deciduous shrubs; indeed, ANPP of graminoids was slightly higher in moderately burned sites than in unburned sites, though not significantly (figure 3 and table 3). By contrast, evergreen shrubs had significantly lower ANPP in the severely burned transects than in moderately burned or unburned transects. Similar to biomass, forb ANPP was not significantly affected by burn severity, but was low and variable.

**Table 3.** Results of analysis of variance on above-ground net primary productivity (ANPP) and N pools in ANPP. Treatments that share the same lower case letter were not significantly different in post-hoc tests (Tukey's HSD) performed when burn severity was significant at *p* < 0.05 (bold). Ndf, numerator degrees of freedom; Ddf, denominator degrees of freedom; U, unburned, M, moderately burned; S, severely burned.

growth form/category	factor								
	burn severity					transect [burn severity]			
	Ndf	Ddf	*F*	*p*	post-hoc	Ndf	Ddf	*F*	*p*
total above-ground net primary productivity (ANPP)	2	54	1.0775	0.3476		3	54	0.7114	0.5494
graminoids	2	54	1.0381	0.3611		3	54	0.7548	0.5244
deciduous shrubs	2	54	2.9568	0.0605		3	54	0.8227	0.4871
evergreen shrubs^a^	2	54	15.7564	**<0.0001**	U*a*; M*a*; > S*b*	3	54	1.5459	0.2132
forbs	2	54	1.1523	0.3235		3	54	0.7814	0.5095
total N pool in ANPP	2	54	1.1746	0.3167		3	54	0.9686	0.4143
graminoids	2	54	1.0111	0.3706		3	54	1.0858	0.3630
deciduous shrubs	2	54	1.9846	0.1473		3	54	0.5714	0.6362
evergreen shrubs^a^	2	54	12.8613	**<0.0001**	U*a*; M*a*; > S*b*	3	54	1.2615	0.2968
forbs	2	54	1.2366	0.2985		3	54	0.6649	0.5772

^a^Data were rank-transformed to meet the assumptions of homogeneity of variance.

### (b) Allocation

To assess whether biomass allocation was altered following fire, we looked at the relationships between root biomass and ANPP or leaf biomass. Fine root biomass explained nearly half the variance in ANPP across all the burned and unburned quadrats (*t* = 5.08, *p* < 0.0001, *r*^2^ = 0.48). By burn severity, the relationship between fine root biomass and ANPP was strong for both unburned (*t* = 4.44, *p* = 0.0022, *r*^2^ = 0.71) and moderately burned (*t* = 3.68, *p* = 0.0062, *r*^2^ = 0.68) sites, but was not significant for severely burned sites. These relationships were driven by fine roots in the organic soil layer; relationships between ANPP and roots in the mineral soil layer were not significant. Fine roots of *E. vaginatum* were responsible for the significant relationship between ANPP and fine root biomass in moderately burned sites, whereas other fine roots were responsible for the significant relationship in unburned sites. Interestingly, the fine-root : leaf–blade ratio for *E. vaginatum* was threefold higher in burned areas, having a mean of 0.56 in unburned transects, 1.66 in moderately burned transects and 1.65 in severely burned transects. This suggests that relative biomass allocation shifted towards roots, at least for this species, during regrowth following the fire.

### (c) Nitrogen in live plant biomass

Nitrogen pools in live biomass and ANPP mostly followed the same overall patterns of statistical significance as seen for biomass and ANPP (figures 2*b*,*d* and 3*b*; tables 3 and 4), because differences in N concentration of plants and their tissue types were small compared with differences in biomass across the burn severity gradient. An exception was that, for deciduous shrubs, the N pool in biomass in moderately burned transects was not statistically different from that in unburned transects, in contrast to biomass, which was significantly lower in moderately burned than in unburned transects (tables 2 and 4). This indicated that deciduous shrubs contained more N per unit of biomass in moderately burned sites than in unburned sites. Also, in comparison with root biomass, root N pools were proportionately lower in moderately burned transects relative to unburned transects (figure 2*b*,*d*), though not enough to be significantly different (tables 2 and 4).

**Table 4.** Results of analysis of variance on N pools in live plant biomass by growth form, and on N pools in live roots. Treatments that share the same lower case letter were not significantly different in post-hoc tests (Tukey's HSD) performed when burn severity was significant at *p* < 0.05 (bold). Ndf, numerator degrees of freedom; Ddf, denominator degrees of freedom; U, unburned; M, moderately burned; S, severely burned.

growth form/category	factor								
	burn severity					transect [burn severity]			
	Ndf	Ddf	*F*	*p*	post-hoc	Ndf	Ddf	*F*	*p*
plant biomass excluding roots^a^	2	54	7.1582	**0.0017**	U*a*; > M*b*; S*b*	3	54	1.4930	0.2268
graminoids	2	54	0.4734	0.6254		3	54	0.8743	0.4602
deciduous shrubs	2	54	5.3330	**0.0077**	U*a*; M*ab*; > S*b*	3	54	0.3228	0.8089
evergreen shrubs^c^	2	54	21.7339	**<0.0001**	U*a*; > M*b*; > S*c*	3	54	1.4762	0.2313
forbs	2	54	1.1619	0.3147		3	54	0.7678	0.5131
all roots^b^	2	24	6.8487	**0.0044**	U*a*; M*ab*; > S*b*	3	24	2.6005	0.0755
all roots from organic soil^b,c^	2	24	25.1263	**<0.0001**	U*a*; M*a*; > S*b*	3	24	2.9088	0.0553
fine roots from organic soil^c^	2	24	19.9035	**<0.0001**	U*a*; M*a*; > S*b*	3	24	2.4241	0.0905
* Eriophorum* roots^c^	2	24	3.3597	0.0517	U*ab*; < M*a*; > S*b*	3	24	1.4676	0.2484
other roots^c^	2	24	30.9733	**<0.0001**	U*a*; > M*b*; > S*c*	3	24	0.5042	0.6830
fine roots from mineral soil	2	24	0.4713	0.6298		3	24	1.5077	0.2379
* Eriophorum* roots	2	24	1.1710	0.3271		3	24	0.3174	0.8127
other roots^c^	2	24	1.0578	0.3628		3	24	1.1856	0.3362
all plant biomass including roots^c^	2	24	11.2055	**0.0004**	U*a*; > M*b*; > S*b*	3	24	1.6339	0.2078

^a^Includes data on moss and lichen biomass, which could not be analysed separately because they did not meet the assumptions of analysis of variance.

^b^Includes coarse roots from organic soil, which could not be analysed separately because there were no coarse roots in the severely burned transects, so the data did not meet the assumptions of analysis of variance.

^c^Data were rank-transformed to meet the assumptions of homogeneity of variance.

**Figure 4.**
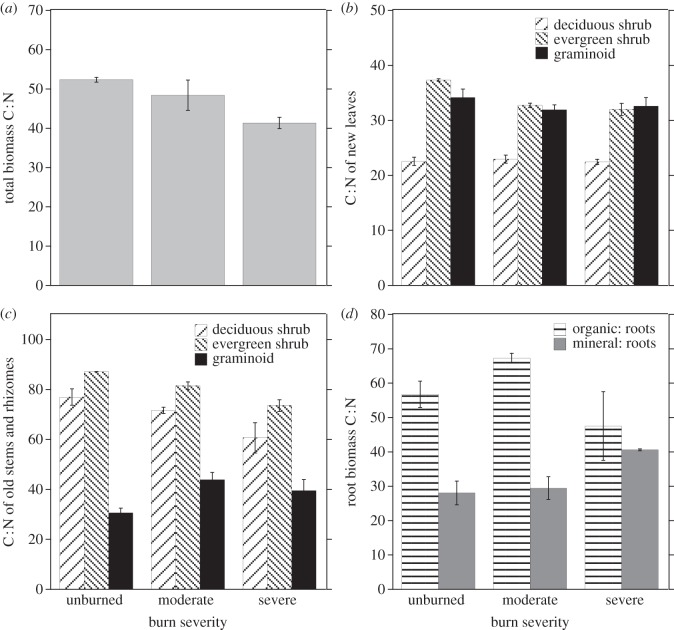
C : N ratios in live biomass for plants from unburned, moderately burned, and severely burned transects. (*a*) C : N of total biomass. (*b*) C : N of new leaves for different growth forms (deciduous shrubs, evergreen shrubs and graminoids). (*c*) C : N of old stems and rhizomes, by growth form. (*d*) C : N of root biomass for roots from the organic horizon and roots from the mineral horizon. Error bars = 1 s.e. for C : N values between transects (*n* = 2).

To assess whether the vegetation in burned sites had changed patterns of nitrogen allocation relative to vegetation in unburned sites, we compared C : N ratios for different tissue types and species. For a given species, tissue type and burn severity, C : N was usually not significantly different among species within a growth form. Overall, the C : N ratio of total live biomass was significantly higher in unburned sites than in severely burned sites, and intermediate in moderately burned sites (figure 4*a* and table 5). Both deciduous and evergreen shrubs had lower C : N ratios in burned sites than in unburned sites, but graminoids showed the opposite pattern, having significantly higher C : N in moderately burned sites than in unburned sites (table 5). For graminoids, this change was caused by a reduction in their overall N : biomass ratio in burned sites, rather than by an increase in their C : biomass ratio, suggesting N stress rather than by greater photosynthetic C accumulation.

**Table 5.** Results of analysis of variance on C : N ratios in live plant biomass by growth form, and in live roots. Treatments that share the same lower case letter were not significantly different in post-hoc tests (Tukey's HSD) performed when burn severity was significant at *p* < 0.05 (bold). Ndf, numerator degrees of freedom; Ddf, denominator degrees of freedom; U, unburned; M, moderately burned; S, severely burned.

growth form/category	factor								
	burn severity					transect [burn severity]			
	Ndf	Ddf	*F*	*p*	Post-hoc	Ndf	Ddf	*F*	*p*
plant biomass excluding roots^a^	2	54	12.3870	**<0.0001**	U*a*; > M*b*; S*b*	3	54	1.6054	0.1988
graminoids	2	50	4.0748	**0.0229**	U*b*; < M*a*; > S*ab*	3	50	0.5359	0.6598
leaves^c^	2	50	1.4717	0.2393		3	50	2.1432	0.1065
rhizomes	2	47	11.0020	**<0.0001**	U*b*; < M*a*; S*a*	3	47	2.3295	0.0864
deciduous shrubs	2	48	5.7693	**0.0057**	U*a*; M*ab*; > S*b*	3	48	0.2333	0.8728
leaves	2	44	0.5303	0.5921		3	44	3.8473	**0.0157**
new stems^c^	2	42	0.1069	0.8988		3	42	3.2670	**0.0305**
old stems and below-ground stems	2	47	11.2960	**<0.0001**	U*a*; M*a*; > S*b*	3	47	2.5931	0.0637
evergreen shrubs^b^	2	51	11.6914	**<0.0001**	U*a*; > M*b*; S*b*	3	51	0.1449	0.9325
new leaves	2	49	7.6107	**0.0013**	U*a*; > M*b*; S*b*	3	49	0.3574	0.7840
new stem	2	49	3.9488	**0.0257**	U*a*; > M*b*; < S*ab*	3	49	1.0423	0.3822
old leaves	2	49	22.8206	**<0.0001**	U*a*; < M*b*; < S*c*	3	49	3.6152	**0.0195**
old stems and below-ground stems	2	51	16.1898	**<0.0001**	U*a*; > M*b*; > S*c*	3	51	0.8932	0.4511
all roots^b^	2	24	5.6843	**0.0095**	U*a*; M*a*; > S*b*	3	24	0.5289	0.6667
all roots from organic soil	2	22	3.9976	**0.0330**	U*ab*; <M*a*; >S*b*	3	22	1.4254	0.2622
fine roots from organic soil	2	22	3.0758	0.0664		3	22	1.5839	0.2217
*Eriophorum* roots	2	10	13.0842	**0.0016**	U*b*; < M*a*; > S*b*	2	10	3.1852	0.0851
other roots	2	10	3.5332	0.0691		2	10	0.0107	0.9894
fine roots from mineral soil	2	21	9.1689	**0.0014**	U*b*; M*b*; < S*a*	3	21	2.2193	0.1158
*Eriophorum* roots	2	10	9.6355	**0.0047**	U*b*; M*b*; < S*a*	2	10	3.7558	0.0607
other roots	2	10	2.2183	0.1595		2	10	15.5170	**0.0009**
all plant biomass including roots	2	24	4.2322	**0.0266**	U*a*; > M*ab*; > S*b*	3	24	0.7866	0.5132

^a^Includes data on moss and lichen biomass, which could not be analysed separately because they did not meet the assumptions of analysis of variance.

^b^Data were rank-transformed to meet the assumptions of homogeneity of variance.

^c^Data could not be transformed to achieve homogeneity of variance, due to high variability in one of the severely burned transects.

Old stems and rhizomes are the most significant storage organs for tundra plants. C : N in old stems was lower in deciduous and evergreen shrubs from burned sites than from unburned sites, which could indicate either greater N retention (perhaps due either to higher uptake or to less use of existing N stores for new growth) or lower C storage (perhaps owing to higher growth demand for stored carbohydrates) in these organs (figure 4*c* and table 5). For both evergreen and deciduous shrubs, both N : biomass and C : biomass ratios were slightly higher in old stems from burned sites, which suggests that the decrease in C : N ratio in burned sites was driven by greater retention of N rather than less retention of C. By contrast, C : N of graminoid rhizomes was higher in burned sites than unburned sites, which could indicate either N stress in burned sites or higher C storage, perhaps from enhanced photosynthetic activity (figure 4*c* and table 5). The N : biomass ratio in the rhizomes of graminoids was lower, and the C : biomass ratio slightly higher in burned sites, compared with unburned sites. Again, this indicates some degree of N stress in graminoids from the burned areas. Moreover, the C : N of both graminoid and deciduous shrub leaves was not significantly different between burned and unburned sites (figure 4*b* and table 5), suggesting that their photosynthetic capacity probably did not differ. C : N was lower in new leaves of evergreen shrubs from burned sites than in unburned sites (figure 4*b* and table 5).

Root C : N was much greater than C : N of stems or leaves. Roots from organic soils had the highest C : N in moderately burned sites, whereas roots from mineral soils had the highest C : N in severely burned sites (table 5). This suggests that most N taken up by plants was not retained in their roots in unburned sites, and was retained even less in roots from burned sites. C : N of fine root biomass increased in both moderately and severely burned sites relative to unburned sites (figure 4*d*), though the difference was significant only for roots from mineral soil (figure 4*d* and table 5). The increased fine root C : N in burned sites was not due solely to a change in the relative abundance of *E. vaginatum* versus other fine roots, because the C : N of *E. vaginatum* roots was also significantly higher in moderately burned sites (table 5).

Together, all of these C : N results suggest that deciduous and evergreen shrubs growing in burned transects had greater stored reserves of N per unit of biomass than in unburned sites, perhaps because their biomass was still lower than in unburned sites. By contrast, graminoids actually had less N reserves per unit of biomass in burned than in unburned sites, though their biomass was similar.

### (d) Nitrogen in soils

**Figure 5.**
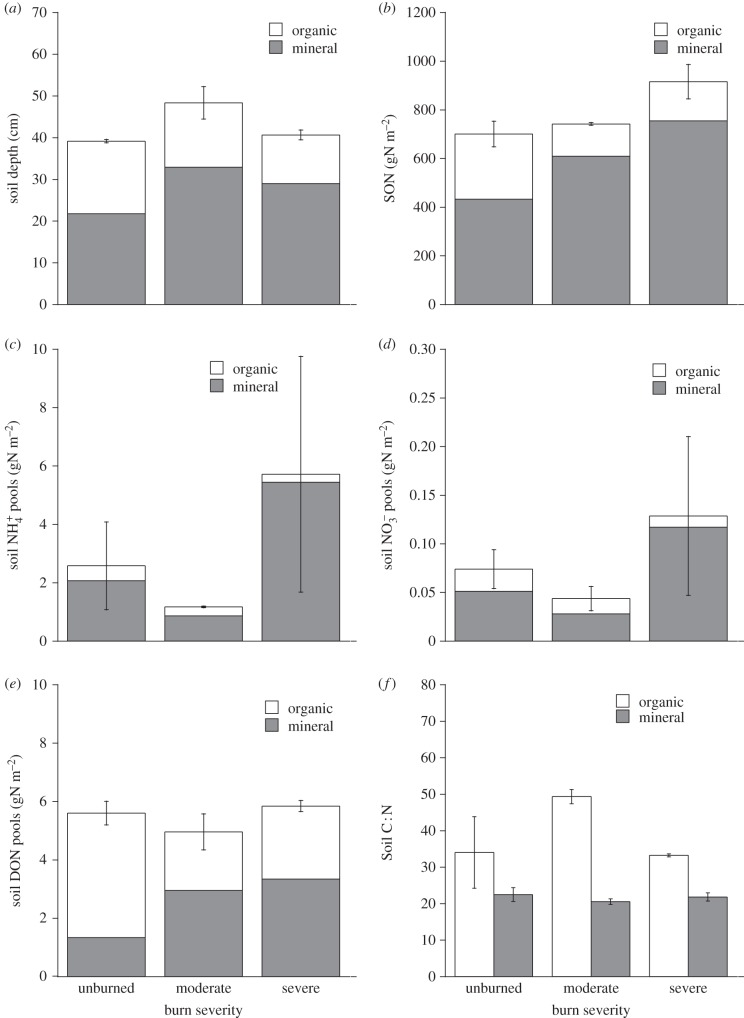
Soil variables from unburned, moderately burned and severely burned transects. (*a*) Depth from the surface to the organic–mineral layer interface (organic), and from the organic–mineral interface to ice (mineral). (*b*) Soil organic N content for the organic and mineral soil layers. (*c*) The mass of N in ammonium extracted from organic and mineral soil layers. (*d*) The mass of N in nitrate extracted from the organic and mineral soil layers. (*e*) The mass of N in dissolved organic N extracted from organic and mineral soil layers. (*f*) C : N of the fine soil fraction. Error bars = 1 s.e. for total pool sizes between transects (*n* = 2).

Although on average, the Anaktuvuk River fire resulted in a loss of 6.1 cm of soil depth [6], there were no significant differences in the depth of the organic soil layer where we sampled (figure 5*a* and table 6). This likely occurred because many of the areas that burned most deeply also had the thickest pre-fire organic layers; the depth of residual organic material is not a good proxy for combustion [6]. By contrast, the depth of the thawed mineral soil layer was significantly greater in burned sites (figure 5*a* and table 6). Bulk soil organic N (SON) of the mineral soil layer (but not the organic soil layer) was higher in burned sites than in unburned sites (figure 5*b* and table 6). This was due to the greater depth of thaw, and thus deeper mineral layer, in burned sites, because the C : N of mineral soil did not differ between burned and unburned sites (figure 5*f* and table 6). However, C : N of organic soils was higher in moderately burned sites than in either unburned or severely burned sites (figure 5*f* and table 6). Plant-available inorganic 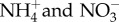 pools did not differ significantly between burned and unburned sites, for either organic or mineral soil layers, although they were quite variable (figure 5*c*,*d* and table 6). Less DON in the organic soil layer occurred in burned sites than in unburned sites, but the opposite pattern prevailed in the mineral soil layer, so that the total soil pool size was about the same (figure 5*e* and table 6). Microbial biomass N did not differ significantly between burned and unburned sites, in either organic or mineral soil layers (table 6). Overall, these results suggest that plant-available soil N summed over organic and mineral soil layers was not higher in burned sites than in unburned sites, by the time of our biomass harvest.

**Table 6.** Results of analysis of variance on soil N pools. All variables were expressed in g N m^−2^ for the entire mineral or organic soil layer, except for depth (cm) and microbial biomass N, which was expressed in gN g^−1^ of oven-dried soil. Treatments that share the same lower case letter were not significantly different in post-hoc tests (Tukey's HSD) performed when burn severity was significant at *p* < 0.05 (bold). Ndf, numerator degrees of freedom; Ddf, denominator degrees of freedom; U, Unburned; M, moderately burned; S, severely burned; SON, soil organic N; DON, dissolved organic N; MB-N, microbial biomass N.

soil variables	factor								
	burn severity					transect [burn severity]			
	Ndf	Ddf	*F*	*p*	post-hoc	Ndf	Ddf	*F*	*p*
organic soil									
depth	2	24	2.0340	0.1528		3	24	0.0995	0.9595
SON	2	24	2.3745	0.1146		3	24	0.3897	0.7615
C : N^a^	2	24	4.2199	**0.0269**	U*b*; <M*a*; > S*b*	3	24	2.1593	0.1191
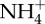	2	24	2.2593	0.1262		3	24	0.7737	0.5201
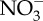	2	24	1.3739	0.2723		3	24	0.5040	0.6832
DON	2	24	4.8540	**0.0170**	U*a*; > M*b*; S*ab*	3	24	0.1638	0.9197
MB-N	2	24	0.3901	0.6812		3	24	0.4652	0.7093
mineral soil									
depth	2	24	4.4887	**0.0221**	U*b*; < M*a*; S*ab*	3	24	0.7418	0.5376
SON	2	24	5.7819	**0.0089**	U*b*; < M*ab*; < S*a*	3	24	0.4452	0.7229
C : N	2	24	2.5115	0.1022		3	24	4.8219	**0.0091**
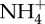 ^a^	2	24	1.5071	0.2418		3	24	2.1852	0.1160
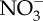 ^a^	2	24	1.9712	0.1612		3	24	4.0024	**0.0192**
DON^b^	2	23	6.1966	**0.0070**	U*b*; < M*a*; S*a*	3	23	1.0211	0.4015
MB-N	2	24	0.3620	0.7000		3	24	0.7372	0.5401

^a^Data were rank-transformed to meet the assumptions of homogeneity of variance.

^b^One high outlier removed prior to analysis.

## 4. Discussion

Despite the high severity of the Anaktuvuk River fire, ANPP of vascular plants in both moderately burned and severely burned tundra had recovered to the unburned level by 4 years following the fire. Vascular plant biomass in the burn still remained below the unburned level. Graminoid biomass was similar in burned sites and in unburned tundra, but deciduous and evergreen shrub biomass had not recovered as completely. Rapid recovery of plant cover and remotely sensed surface properties, such as albedo and vegetation indices, as well as an early, vigorous growth response by resprouting graminoids have been reported after several other tundra fires [10,13,40]. The much greater changes we observed after fire in the biomass and composition of the non-vascular, compared with the vascular, plant community, have also been seen after other tundra fires; lichen biomass, in particular, appears to take decades to centuries to recover from the disturbances of fires or heavy overgrazing by reindeer, especially under a warming climate [11,12,16,41,42]. However, initial increases in the relative abundance of graminoids relative to shrubs are often transient, and over two to three decades, tussock tundra vegetation can return to a post-fire composition that is similar to that of unburned tundra except for the lichen component [12,15], showing resilience to this disturbance. This is a possible outcome over much of the area burned in the Anaktuvuk River fire.

In the boreal forest, fire severity affects community assembly and secondary succession primarily by influencing the relative success of different plant regeneration strategies [43]. Also, tree seedlings mostly establish within the first decade following fire, which sets the trajectory for canopy dominance [44,45]. In burned black spruce forests, succession after low-to-moderate severity fires is characterized by regrowth from species capable of resprouting following fire and establishment of black spruce seedlings, which returns the burned area to a black spruce forest of similar vegetation composition [43,45]. By contrast, establishment of colonizers and deciduous tree seedlings is much more important in high severity fires, where consumption of thick organic layers provides a better substrate for seed germination and seedling survival, and leads to alternative trajectories of succession dominated by deciduous trees [43,45]. Many tundra species are also found in the boreal forest understory, and virtually all the vascular plant species we observed in the area burned by the Anaktuvuk River fire resprouted following the fire, even in severely burned transects. Although the Anaktuvuk River fire was unusually severe for a tundra fire, from the abundance of resprouters in burned areas it appears to be more similar to moderate than to severe boreal forest fires. The plant community assembling in our burned sites is from the same species pool as in the unburned sites, because all the vascular plant species in burned areas were also seen in unburned tundra. If tundra follows similar successional pathways as seen in boreal forest (without the trees), then the vegetation in the burned areas we studied may be resilient to this disturbance and return to its pre-fire composition.

Another factor that could promote resilience in vegetation recovery following fire in tundra is low soil nutrient availability, because a limited number of species have conservative traits that allow them to succeed under nutrient limitation, and ANPP in unburned tussock tundra has been shown to be strongly nutrient-limited [21–23]. Total vascular ANPP (200 g m^−2^ yr^−1^) of unburned tundra in the vicinity of the Anaktuvuk River fire is remarkably similar to other recent measurements from sites near Toolik Lake (e.g. 180 g m^−2^ yr^−1^ [34]; 220 g m^−2^ yr^−1^ [23]), even though the Anaktuvuk River fire sites are nearly 300 m lower in elevation, and mean summer air temperatures were 0.5–1.4°C warmer, between 2008 and 2010, than at Toolik Lake [46,47]. This suggests that the ANPP of tundra that we measured at the unburned sites near the Anaktuvuk River fire scar is just as nutrient-limited as it is in tundra near Toolik Lake, despite a more favourable climate during the growing season. The Anaktuvuk River fire scar lies north of the drift limits of the Itkillik I glaciation [48], and thus has been deglaciated for considerably longer than sites around Toolik Lake (which lie within the limits of the Itkillik I drift, and were glaciated approx. 50 000 years BP [49]). The older landscape of the Anaktuvuk River fire has had more time for weathering and paludification to occur, which could lead to low nutrient availability (although soil pH values were similar to those found in moist acidic tundra at Toolik Lake [50]). Starting from this nutrient-limited state, combustion during the Anaktuvuk River fire removed an estimated 400 years of N accumulation [6], which suggests that this landscape might be expected to have even lower soil nutrient availability now.

Despite this, the rapid regrowth of the vegetation and the lowered C : N ratio in old stems of deciduous and evergreen shrubs suggest that soil N was not limiting to plants immediately following the Anaktuvuk River fire, perhaps because of reduced competition for soil N after most of the above-ground plant biomass had been consumed by combustion. Indeed, across all the permanent transects, the relative availability of inorganic N and P, as assessed by ion-exchange resin bags, was approximately two to three times higher in burned sites than in unburned sites during 2008–2009 (data not shown). However, by the time of our biomass harvest, plant-available soil N pools did not differ significantly between burned and unburned sites, suggesting that nutrient limitation is now occurring. Similarly, transient increases in soil N availability lasting 1–2 years have been seen following stand-replacing forest fires [25].

Changes in plant allocation also suggest nutrient limitation at the time of our harvest. The increased fine-root : leaf-blade ratios in *E. vaginatum* suggest that in burned sites this species had to allocate more resources towards acquiring N than in unburned sites (*E. vaginatum* grows an entirely new set of roots each year). *Eriophorum vaginatum* appears to be fairly flexible in its relative allocation to fine roots [51], and adjusts its fine-root : leaf-blade ratio downward under fertilization when N is readily available [52]. The increased C : N ratio in graminoid biomass that we observed in burned sites also suggests that N stress may now be occurring in these plants after their initial, vigorous growth spurt following the fire.

Over the longer term, it seems likely that low nutrient availability will confer resilience on this vegetation and constrain it from shifting to another successional trajectory following fire, such as to dominance by large deciduous shrubs, unless there is persistent thawing of permafrost. Thawing of permafrost results in changed hydrology and persistently higher nutrient availability, and promotes a shift from dominance by graminoids to dominance by deciduous shrubs in tundra [53]. In two cases, vegetation has also been seen to shift from tussock tundra to dominance by large deciduous shrubs two decades after fire [11,16]. In one, deciduous shrub abundance increased on hillslope sites that were better drained and probably had a deeper active layer relative to other tundra fire sites [11]. In the other case, the transition occurred in a severely burned area close to treeline, where the active layer was probably deeper than in more northern sites, due both to an intrinsically warmer climate, and to having experienced a period of warming and drying during the 22 years following the fire [16]. Long-term (more than 30 years) fertilization experiments that resulted in the development of persistent shrub dominance over several decades went through a transient phase of increased graminoid abundance in the first 3–5 years, and seem to require a period of prolonged high nutrient availability for deciduous shrubs to achieve dominance [21–23]. Although the landscape of the Anaktuvuk River fire shows no sign yet of heading towards such a transition, its future probably depends mainly on what happens to permafrost and soil nutrient availability over the longer term.

Continuous permafrost underlies the tundra ecosystems of the North Slope of Alaska, and currently only a shallow layer of soil (the active layer) near the surface thaws seasonally. However, recent climate warming at high latitudes has resulted in warming and thawing of permafrost in many regions [54,55]. Over the next century, climate warming will likely interact with fire to accelerate permafrost thaw. Owing to changes in surface energy balance, the depth of the active layer is commonly increased in the first few years following fire in tundra ecosystems [10,13,40,47,56]. After fire, the depth of the active layer in burned sites may be deeper than in unburned sites for two or three decades [14,15,40], though in other cases it returns to unburned levels within about 10 years [13]. It has been suggested that resilience of permafrost to climate change is promoted by negative feedbacks from ecosystem properties that develop during vegetation succession, including canopy effects on shading and snow interception, litterfall quantity and quality, moss growth, increasing moisture, and decreasing soil temperatures that reduce decomposition [57]. In addition, the widespread peat layer that blankets the North Slope of Alaska inhibits large-scale soil erosion and stabilizes permafrost [58]. By contrast, surface water impounded when the ground settles after thawing of subsurface ice can warm ground temperatures by up to 10°C, and accelerate permafrost thaw by interacting with subsurface ice, even in cold climates [57]. Tussock tundra is generally underlain by deep, continuous permafrost, and thus far, recovery of tundra vegetation and permafrost has occurred following most of the tundra fires that have been studied. Indeed, the difference in active layer depth between burned and unburned sites within the Anaktuvuk River fire has already started to decrease [47], although multiple local ground subsidences caused by melting of subsurface ice have been observed within the fire perimeter, mostly on hillslopes [30,58]. For the past few centuries, even in the warmer boreal forest of the Alaskan Interior, permafrost has usually recovered after fire as the forest vegetation and organic soils have built back up, though permafrost there may not be able to recover after fire in the future if the climate continues to warm [57].

It has been suggested that fire may play an important role in maintaining the long-term persistence of tussock tundra, promoting the growth of *E. vaginatum* tussocks by reducing competition from deciduous and evergreen shrubs, and allowing opportunities for seedling establishment [13]. Most of the area of the Anaktuvuk River fire was classified as mixed shrub–sedge tussock tundra, with a shrub cover greater than 25 per cent [30], which is shrubbier than classic tussock tundra [29]. Fire promotes flowering of *E. vaginatum* and establishment of seedlings within one to a few years after fire, though few of these seedlings successfully recruit into the tussock population over the longer term [10,13]. We saw numerous graminoid seedlings in our harvest, but very few dicotyledonous seedlings, as in previous studies, and did not detect any non-native species. The Anaktuvuk River fire scar is located far from any roads that could serve as a source for introducing non-native species; this could allow any disequilibrium between potential flora and climate to persist [59]. That shrub biomass is increasing and shrub ANPP has already reached pre-fire levels suggests that shrubs will return to their former abundance eventually, but a transition to shrub dominance is unlikely as long as permafrost in the burned areas does not thaw deeply.

In conclusion, vegetation recovery after the Anaktuvuk River fire is consistent with what has been observed after other tundra fires from the late-twentieth century, despite the unusual severity and size of this fire. Although the area burned by this fire showed no evidence of burning in the past 5000 years [27], the plant species that occupy the fire scar are common in Alaska, and occur in both tundra [4,13,60] and boreal forest that burns much more frequently. Indeed, they show many characteristics consistent with adaptation to fire [13]. Nitrogen limitation and the persistence of resprouting plants in the burned areas are likely to constrain the successional trajectory back towards mixed shrub–sedge tussock tundra and promote resilience, unless climate warming leads to much greater permafrost thaw, and thus to higher nutrient availability than has yet been seen. Although it is likely that, in the next century, climate warming will interact with fire to enhance permafrost degradation and vegetation change, the Anaktuvuk River fire does not seem to have pushed its landscape over a tipping point into a new regime [9]. Over much of the area of the Anaktuvuk River fire scar, it is likely that the vegetation will be similar to what was there before the fire.

## References

[1] ACIA 2004 Impacts of a warming Arctic: Arctic climate impact assessment. Cambridge, UK: Cambridge University Press

[2] Serreze MC 2000 Observational evidence of recent change in the northern high-latitude environment. Clim. Change 46, 159–207 (doi:10.1023/A:1005504031923)

[3] Kasischke ES Turetsky MR 2006 Recent changes in the fire regime across the North American boreal region: spatial and temporal patterns of burning across Canada and Alaska. Geophys. Res. Lett. 33, L09703 (doi:10.1029/2006GL025677)

[4] Racine CH Dennis JG Patterson WA III 1985 Tundra fire regimes in the Noatak Watershed, Alaska: 1956–1983. Arctic 38, 194–200

[5] Hinzman LD 2005 Evidence and implications of recent climate change in northern Alaska and other Arctic regions. Clim. Change 72, 251–298 (doi:10.1007/s10584-005-5352-2)

[6] Mack MC Bret-Harte MS Hollingsworth TN Jandt RR Schuur EAG Shaver GR Verbyla DL 2011 Carbon loss from an unprecedented Arctic tundra wildfire. Nature 475, 489–492 (doi:10.1038/nature10283) 21796209 10.1038/nature10283

[7] Field CB Lobell DB Peters HA Chiarello NR 2007 Feedbacks of terrestrial ecosystems to climate change. Annu. Rev. Environ. Resour. 32, 1–29 (doi:10.1146/annurev.energy.32.053006.141119)

[8] Schuur EAG*.* 2008 Vulnerability of permafrost carbon to climate change: implications for the global carbon cycle. BioScience 58, 701–714 (doi:10.1641/B580807)

[9] Folke C Carpenter S Walker B Scheffer M Elmqvist T Gunderson L Holling CS 2004 Regime shifts, resilience, and biodiversity in ecosystem management. Annu. Rev. Ecol. Syst. 35, 557–581 (doi:10.1146/annurev.ecolsys.35.021103.105711)

[10] Wein RW Bliss LC 1973 Changes in Arctic *Eriophorum* tussock communities following fire. Ecology 54, 845–852 (doi:10.2307/1935679)

[11] Racine C Jandt R Meyers C Dennis J 2004 Tundra fire and vegetation change along a hillslope on the Seward Peninsula, Alaska, USA. Arctic Antarctic Alpine Res. 36, 1–10 (doi:10.1657/1523-0430(2004)036[0001:TFAVCA]2.0.CO;2)

[12] Jandt R Joly K Meyers CR Racine C 2008 Slow recovery of lichen on burned caribou winter range in Alaska tundra: potential influences of climate warming and other disturbance factors. Arctic Antarctic Alpine Res. 40, 89–95 (doi:10.1657/1523-0430(06-122)[JANDT]2.0.CO;2)

[13] Racine CH Johnson LA Viereck LA 1987 Patterns of vegetation recovery after tundra fires in northwestern Alaska, USA. Arctic Alpine Res. 19, 461–489 (doi:10.2307/1551412)

[14] Fetcher N Beatty TF Mullinax B Winkler DS 1984 Changes in Arctic tussock tundra thirteen years after fire. Ecology 65, 1332–1333 (doi:10.2307/1938338)

[15] Vavrek MC Fetcher N McGraw JB Shaver GR Chapin FS III Bovard B 1999 Recovery of productivity and species diversity in tussock tundra following disturbance. Arctic Antarctic Alpine Res. 31, 254–258 (doi:10.2307/1552254)

[16] Landhausser SM Wein RW 1993 Postfire vegetation recovery and treeline establishment at the Arctic treeline: climate-change–vegetation-response hypotheses. J. Ecol. 81, 665–672 (doi:10.2307/2261664)

[17] Barrett K Rocha AV van de Weg MJ Shaver G 2012 Vegetation shifts observed in Arctic tundra 17 years after fire. Remote Sens. Lett. 3, 729–736 (doi:10.1080/2150704X.2012.676741)

[18] Sturm M Racine C Tape K 2001 Increasing shrub abundance in the Arctic. Nature 411, 546–547 (doi:10.1038/35079180) 11385559 10.1038/35079180

[19] Tape K Sturm M Racine C 2006 The evidence for shrub expansion in Northern Alaska and the Pan-Arctic. Glob. Change Biol. 12, 1–17 (doi:10.1111/j.1365-2486.2006.01128.x)

[20] Higuera PE Brubaker LB Anderson PM Brown TA Kennedy AT Hu FS 2008 Frequent fires in ancient shrub tundra: implications of paleorecords for Arctic environmental change. PLoS ONE 3, e0001744 (doi:10.1371/journal.pone.0001744) 18320025 10.1371/journal.pone.0001744 PMC2254503

[21] Chapin FS III Shaver GR Giblin AE Nadelhoffer KJ Laundre JA 1995 Response of Arctic tundra to experimental and observed changes in climate. Ecology 76, 694–711 (doi:10.2307/1939337)

[22] Shaver GR Bret-Harte MS Jones MH Johnstone J Gough L Laundre J Chapin FS III 2001 Species changes interact with fertilizer addition to control 15 years of change in tundra. Ecology 82, 3163–3181 (doi:10.1890/0012-9658(2001)082[3163:SCIWFT]2.0.CO;2)

[23] Mack MC Schuur EAG Bret-Harte MS Shaver GR Chapin FS III 2004 Ecosystem carbon storage in Arctic tundra reduced by long-term nutrient fertilization. Nature 431, 440–443 (doi:10.1038/nature02887) 15386009 10.1038/nature02887

[24] Walker MD 2006 Plant community responses to experimental warming across the tundra biome. Proc. Natl Acad. Sci. USA 103, 1342–1346 (doi:10.1073/pnas.0503198103) 16428292 10.1073/pnas.0503198103 PMC1360515

[25] Smithwick EAH Turner MG Mack MC Chapin FS III 2005 Postfire soil N cycling in northern conifer forests affected by severe, stand-replacing wildfires. Ecosystems 8, 163–181 (doi:10.1007/s10021-004-0097-8)

[26] Jones BM Kolden CA Jandt RR Abatzoglou JT Urban F Arp CD 2009 Fire behavior, weather, and burn severity of the 2007 Anaktuvuk River tundra fire, North Slope, Alaska. Arctic Antarctic Alpine Res. 41, 309–316 (doi:10.1657/1938-4246-41.3.309)

[27] Hu FS Higuera PE Walsh JE Chapman WL Duffy PA Brubaker LB Chipman ML 2010 Tundra burning in Alaska: linkages to climatic change and sea ice retreat. J. Geophys. Res. 115, G04002 (doi:10.1029/2009JG001270)

[28] Bliss LC Matveyeva NV 1992 Circumpolar Arctic vegetation. In Arctic ecosystems in a changing climate: an ecophysiological perspective (eds Chapin FS III Jefferies RL Reynolds JF Shaver GR Svoboda J), pp. 59–89 San Diego, CA: Academic Press

[29] Viereck LA Dyrness CT Batten AR Wenzlick KJ 1992 The Alaska vegetation classification: US Forest Service, Pacific Northwest Research Station. Report no. PNW-GTR-286

[30] Jandt RR Miller EA Yokel DA Bret-Harte MS Mack MC Kolden CA 2012 Findings of Anaktuvuk River fire recovery study. Fairbanks, AK: US Bureau of Land Management

[31] Shaver GR Chapin FS III 1991 Production : biomass relationships and element cycling in contrasting Arctic vegetation types. Ecol. Monogr. 61, 1–31 (doi:10.2307/1942997)

[32] Alaska Interagency Fire Effects Task Group 2007 Fire effects monitoring protocol - version 1.0 (includes data sheet templates). Anchorage, AK: Alaska Interagency Fire Effects Task Group

[33] Boelman NT Rocha AV Shaver GR 2011 Understanding burn severity sensing in Arctic tundra: exploring vegetation indices, suboptimal assessment timing and the impact of increasing pixel size. Int. J. Remote Sens. 32, 7033–7056 (doi:10.1080/01431161.2011.611187)

[34] Bret-Harte MS Mack MC Goldsmith GR Sloan D DeMarco J Shaver GR Ray PM Biesinger Z Chapin FS III 2008 Plant functional types do not predict biomass responses to removal and fertilization in Alaskan tussock tundra. J. Ecol. 96, 713–726 (doi:10.1111/j.1365-2745.2008.01378.x) 18784797 10.1111/j.1365-2745.2008.01378.x PMC2438444

[35] Bret-Harte MS Shaver GR Chapin FS III 2002 Primary and secondary growth in Arctic shrubs: implications for community response to environmental change. J. Ecol. 90, 251–267 (doi:10.1046/j.1365-2745.2001.00657.x)

[36] Brookes PD Landman A Pruden G Jenkinson DS 1985 Chloroform fumigation and the release of soil nitrogen: a rapid direct extraction method to measure microbial biomass nitrogen in soil. Soil Biol. Biochem. 17, 837–842 (doi:10.1016/0038-0717(85)90144-0)

[37] JMP 2003 JMP statistical software, 5.0.1.2 edn Cary: NC SAS Institute

[38] Zar JH 1999 Biostatistical analysis, 4th edn New Jersey, NJ: Prentice-Hall

[39] Hoenig JM Heisey DM 2001 The abuse of power: the pervasive fallacy of power calculations for data analysis. Am. Stat. 55, 19–24 (doi:10.1198/000313001300339897)

[40] Rocha AV 2012 The footprint of Alaskan tundra fires during the past half-century: implications for surface properties and radiative forcing. Environ. Res. Lett. 7, 044039 (doi:10.1088/1748-9326/7/4/044039)

[41] Klein DR Shulski M 2009 Lichen recovery following heavy grazing by reindeer delayed by climate warming. AMBIO 38, 11–16 (doi:10.1579/0044-7447-38.1.11) 19260341 10.1579/0044-7447-38.1.11

[42] Joly K Jandt RR Klein DR 2009 Decrease of lichens in Arctic ecosystems: the role of wildfire, caribou, reindeer, competition and climate in north-western Alaska. Polar Res. 28, 433–442 (doi:10.1111/j.1751-8369.2009.00113.x)

[43] Hollingsworth TN Johnstone JF Bernhardt EL Chapin FS III 2013 Fire severity filters regeneration traits to shape community assembly in Alaska's boreal forest. PLoS ONE 8, e56033 (doi:10.1371/journal.pone.0056033) 23418503 10.1371/journal.pone.0056033 PMC3572144

[44] Johnstone JF Chapin FS III Foote J Kemmett S Price K Viereck L 2004 Decadal observations of tree regeneration following fire in boreal forests. Can. J. Forest Res. 34, 267–273 (doi:10.1139/x03-183)

[45] Johnstone JF Hollingsworth TN Chapin FS III Mack MC 2010 Changes in fire regime break the legacy lock on successional trajectories in Alaskan boreal forest. Glob. Change Biol. 16, 1281–1295 (doi:10.1111/j.1365-2486.2009.02051.x)

[46] Environmental Data Center Team 2012 Meteorological monitoring program at Toolik, Alaska. Fairbanks, AK: Toolik Field Station, Institute of Arctic Biology, University of Alaska, Fairbanks. (see http://toolik.alaska.edu/edc/abiotic_monitoring/data_query.php)

[47] Rocha AV Shaver GR 2011 Postfire energy exchange in Arctic tundra: the importance and climatic implications of burn severity. Glob. Change Biol. 17, 2831–2841 (doi:10.1111/j.1365-2486.2011.02441.x)

[48] Hamilton TD Porter SC 1975 Itkillik glaciation in the Brooks Range, northern Alaska. Quat. Res. 5, 471–497 (doi:10.1016/0033-5894(75)90012-5)

[49] Hamilton TD 2002 Glacial geology of Toolik Lake and the upper Kuparuk River region. Fairbanks, AL: Alaska Geobotany Center, Institute of Arctic Biology, University of Alaska, Fairbanks

[50] Hobbie SE Gough L 2002 Foliar and soil nutrients in tundra on glacial landscapes of contrasting ages in northern Alaska. Oecologia 131, 453–462 (doi:10.1007/s00442-002-0892-x) 10.1007/s00442-002-0892-x 28547718

[51] Kummerow J Ellis B 1984 Temperature effect on biomass production and root/shoot biomass ratios in two Arctic sedges under controlled environmental conditions. Can. J. Bot. 62, 2150–2153 (doi:10.1139/b84-294)

[52] Shaver GR Chapin FS III Gartner BL 1986 Factors limiting seasonal growth and peak biomass accumulation in *Eriophorum vaginatum* in Alaskan tussock tundra. J. Ecol. 74, 257–278 (doi:10.2307/2260362)

[53] Schuur EAG Crummer KG Vogel JG Mack MC 2007 Plant species composition and productivity following permafrost thaw and thermokarst in Alaskan tundra. Ecosystems 10, 280–292 (doi:10.1007/s10021-007-9024-0)

[54] Romanovsky VE Osterkamp TE 1997 Thawing of the active layer on the coastal plain of the Alaskan Arctic. Permafrost Periglacial Proc. 8, 1–22 (doi:10.1002/(SICI)1099-1530(199701)8:1<1::AID-PPP243>3.0.CO;2-U)

[55] Jorgensen MT Shur YL Pullman ER 2006 Abrupt increase in permafrost degradation in Arctic Alaska. Geophys. Res. Lett. 33, L02503 (doi:10.1029/2005GL024960)

[56] Liljedahl A Hinzman L Busey R Yoshikawa K 2007 Physical short-term changes after a tussock tundra fire, Seward Peninsula, Alaska. J. Geophys. Res. 112, F02S07 (doi:10.1029/2006JF000554)

[57] Jorgenson MT Romanovsky V Harden J Shur Y O'Donnell J Schuur EAG Kanevsky M Marchenko S 2011 Resilience and vulnerability of permafrost to climate change. Can. J. Forest Res. 40, 1219–1236 (doi:10.1139/X10-060)

[58] Mann DH Groves P Reaner RE Kunz ML 2010 Floodplains, permafrost, cottonwood trees, and peat: what happened the last time climate warmed suddenly in Arctic Alaska? Quat. Sci. Rev. 29, 3812–3830 (doi:10.1016/j.quascirev.2010.09.002)

[59] Normand S 2013 A greener Greenland? Climatic potential and long-term constraints on future expansions of trees and shrubs. Phil. Trans. R. Soc. B 368, 20120479 (doi:10.1098/rstb.2012.0479) 23836785 10.1098/rstb.2012.0479 PMC3720052

[60] Higuera PE Chipman ML Barnes JL Urban MA Hu FS 2011 Variability of tundra fire regimes in Arctic Alaska: millenial scale patterns and ecological implications. Ecol. Appl. 21, 3211–3226 (doi:10.1890/11-0387.1)

